# Oxytocin in the Circadian Timing of Birth

**DOI:** 10.1371/journal.pone.0000922

**Published:** 2007-09-26

**Authors:** Jeffrey Roizen, Christina E. Luedke, Erik D. Herzog, Louis J. Muglia

**Affiliations:** 1 Department of Pediatrics, Washington University School of Medicine, St. Louis, Missouri, United States of America; 2 Department of Molecular Biology and Pharmacology, Washington University School of Medicine, St. Louis, Missouri, United States of America; 3 Department of Obstetrics and Gynecology, Washington University School of Medicine, St. Louis, Missouri, United States of America; 4 Division of Endocrinology, Children's Hospital, Boston, Massachusetts, United States of America; 5 Department of Biology, Washington University, St Louis, Missouri, United States of America; University of Edinburgh, United Kingdom

## Abstract

**Background:**

The molecular components determining the timing for birth remain an incompletely characterized aspect of reproduction, with important conceptual and therapeutic ramifications for management of preterm, post-term and arrested labor.

**Methodology/Principal Findings:**

To test the hypothesis that oxytocin mediates circadian regulation of birth, we evaluated parturition timing following shifts in light cycles in oxytocin (OT)-deficient mice. We find that, in contrast to wild type mice that do not shift the timing of birth following a 6-h advance or delay in the light cycle, OT-deficient mice delivered at random times of day. Moreover, shifts in the light-dark cycle of gravid wild type mice have little impact on the pattern of circadian oxytocin release.

**Conclusions/Significance:**

Our results demonstrate oxytocin plays a critical role in minimizing labor disruption due to circadian clock resetting.

## Introduction

The clock metering the duration of pregnancy has been suggested to consist of two interacting timers; an interval timer measuring the overall length of gestation, and a circadian timer defining when within a 24-hour cycle birth takes place [1]. All animals studied to date (including hamsters, rats, mice, sheep, and humans) exhibit a robust circadian rhythmicity for the onset of labor, though this timing does not correlate with either active or inactive periods or position in a light-dark cycle. The circadian timing for birth depends upon the hypothalamic suprachiasmatic nucleus (SCN) as demonstrated by lesion studies in rodents [2]. The interval timer, however, appears independent of the SCN, as lesioned animals deliver on average at the appropriate duration of gestation [3], [4].

One particularly attractive signaling pathway by which the SCN could communicate to the periphery to control parturition is via the hypothalamic nonapeptide oxytocin. Highly conserved patterns of oxytocin secretion and oxytocin receptor induction have been demonstrated amongst mammals. Therefore, it was surprising that oxytocin-deficient mice had no abnormalities in the overall duration or progression of labor [5], [6]. These studies suggest that oxytocin does not play an indispensable role in the interval timer of birth. Given the strong evolutionary conservation of oxytocin regulation during pregnancy, and its known circadian release in virgin and gravid animals [7], [8], we hypothesized that oxytocin instead mediates circadian gating of birth. To test this hypothesis, we evaluated the effects shifts in a 12-h light-12-h dark cycle on the timing of parturition in oxytocin-deficient mice.

## Results

**Figure 1:**
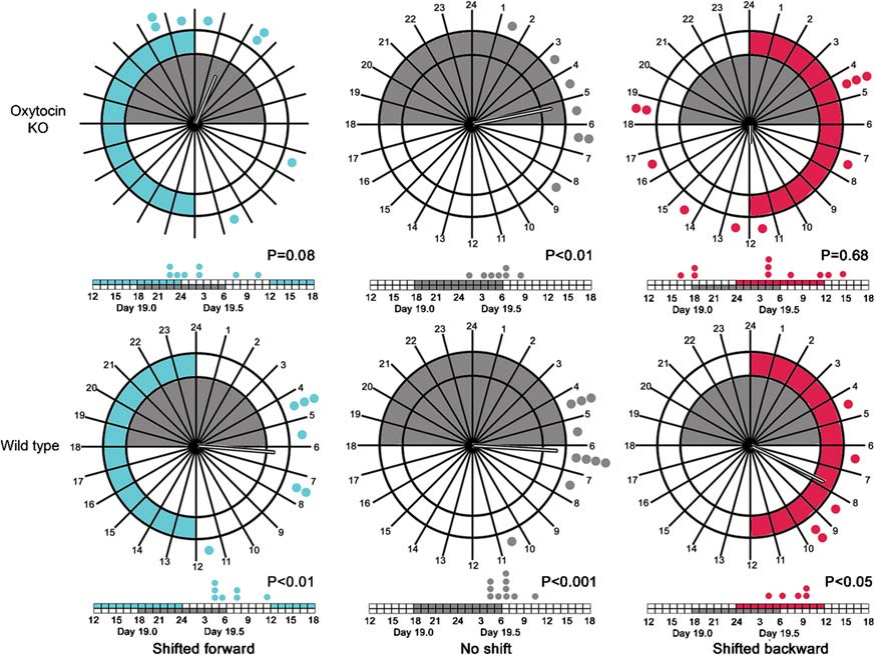
Distribution of births over circadian clock time. Top row, OT Knockout Mice; Bottom Row, Wild Type Mice. Left column, shifted forward; middle column no shift, right column, Shifted back. Gray Shading, normal night; blue shading, forward shifted night; red shading, backward shifted night. On the clock, each dot represents one birth of the mice in that group, the vector emanating from the radius in each clock represents by direction the average time of birth and by magnitude, the strength of that average. Below each circadian clock is a linear timeline of births, with each dot representing a single labor from mice in that group. Each segment represents one hour. For each clock, the P value indicates the likelihood of the observed clustering of births if the births are randomly distributed.

On the morning of day post-coitum (dpc) 4.5, pregnant mice were divided into three groups (shifted forward 6 hours, unshifted and shifted backward 6 hours). Both wild type (WT) and oxytocin knockout (OT KO) mice fully adjusted their locomotor activity onsets with 6 days of the advanced schedule and 4 days of the delayed schedule (data not shown). Surprisingly, the shifted light cycles failed to shift significantly the absolute timing of labor in WT mice (Fig. 1; shifted forward P<0.01, n = 7; unshifted P<0.001 n = 10; shifted backward P<0.05, n = 5, Rayleigh's Test). The unshifted OT KO mice exhibited a similar time and distribution of labor as the WT mice. In contrast, the onset of labor in OT KO mice shifted forward or backward was distributed randomly with respect to time of day (shifted forward vector magnitude 0.56, n = 8, P = 0.08, n = 8; shifted backward vector magnitude 0.20, n = 10, P = 0.68). Furthermore, in the backward shifted OT KO mice, two mice died due to failed labor. Thus, in the absence of a shift, labor timing in OT KO mice is indistinguishable from that in WT mice, however either shift causes the OT KO mice to lose the circadian distribution of labor.

**Figure 2:**
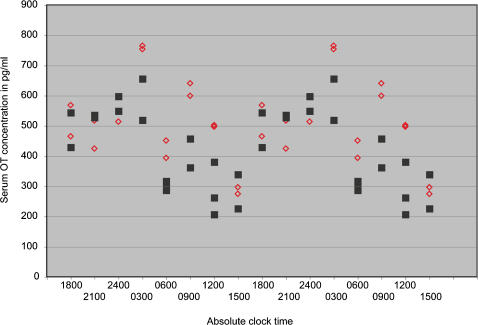
Circadian serum oxytocin rhythm does not differ between backward-shifted and unshifted wild type mice over the 24 period of dpc 17.5. Backward shift; red diamonds, Unshifted; black squares. Each point represents the average of duplicate measurements on plasma from a single mouse, and is double-plotted to show the rhythmic pattern.

To further understand the mechanism by which OT contributes to maintaining the duration of gestation and circadian modulation of birth timing in the face of altered light cycles, we measured plasma OT concentration in WT mice that had been subjected to a backward shift of the light cycle or remained unshifted in the original light cycle (Fig. 2). We hypothesized that, while locomotor activity shifted, the circadian pattern of serum OT rhythms would not change as a function of a shift in the light cycle. This maintenance of OT rhythmicity would facilitate the circadian gating of birth in WT mice. We analyzed the pattern of serum OT concentrations beginning on dpc 17.5 for a 24 h cycle. We chose this point because it was well after the change in locomotor activity after a shift in the light cycle had been established, and before changes in OT concentration associated with active labor would occur. We found that oxytocin levels were rhythmic in the unshifted mice (peak-to-trough amplitude of 260 pg/ml, P = 0.03 by COSOPT [9]), and higher amplitude, but less rhythmic in shifted mice (peak-to-trough amplitude of 292 pg/ml, P = 0.07). Shifting the light cycle left the oxytocin rhythm largely intact except for an increase in a smaller, secondary daily peak. This finding suggests that the major daily peak in plasma oxytocin does not shift with the light cycle or locomotor behavior during pregnancy.

## Discussion

Three significant implications arise from these results. First, from the data presented, it is expected that the pattern of OT secretion is fixed early in gestation. This oxytocin rhythm does not shift with changes in the light cycle (as in transmeridian travel or shift work) during gestation indicating that it is controlled by a circadian pacemaker distinct from the master clock that regulates locomotor activity. This unknown pacemaker is relatively insensitive to changes in the light cycle during gestation. The dissociation between the circadian shift in locomotor rhythm and the absence of a shift in serum oxytocin rhythm is surprising. While oxytocin is not synthesized in the SCN, oxytocin neurons in the paraventricular nucleus of the hypothalamus are targets of vasoactive intestinal peptide secreting neurons of the suprachiamatic nucleus [10], [11]. Moreover, bidirectional signaling has been demonstrated between the SCN and supraoptic nucleus, another site of oxytocin synthesis destined for systemic secretion [12]. How the SCN–oxytocin neuronal signals are uncoupled during pregnancy remains to be elucidated.

Second, the circadian pattern of oxytocin regulation is a primary determinant of the circadian timing of labor. We draw this conclusion from the observations that serum OT rhythms do not shift in WT mice when comparing levels from mice that have undergone a shift in their light cycle early in pregnancy with those that have not, and the timing for delivery in WT mice does not change with a shift in the light cycle while this timing becomes random in OT deficient mice. This endogenous rhythm in OT rather than environmental cues from light or darkness determines the hour of birth as supporting observations form seminal studies of photoperiod effects on parturition [13]. Since we placed mice in constant darkness at dpc 17.5, it remains unknown as to whether continuing the light-dark cycle would alter the timing changes we observed in the OT KO mice. The pattern of serum OT regulation we measure differs somewhat from previous measures in rats in that the rise we observe occurs closer to the onset of the dark phase of the light cycle and extends for a longer duration into the dark phase [8], [10]. This difference may arise from changes in regulation in mouse pregnancy or because of the strain we used, C57BL/6, that lacks melatonin, a regulator of oxytocin rhythmicity [14].

Third, in the absence of oxytocin, the circadian gating of birth is lost following a shift in the light cycle so that parturition has a much greater chance of being dysfunctional. This final implication is supported by the labor phenotype of circadian mutant mice [3]. Further, as oxtyocin has both actions that delay the onset of labor by maintaining luteal function or promote labor by augmenting uterine contractions, the circadian release would enhance tissue-specific peripheral changes in OT sensitivity to focus the timing and progression of labor [15]. Thus, we find OT plays a critical role in minimizing disruption of labor in the face of resetting the master circadian clock.

## Materials and Methods

### Creation of a null allele for Oxytocin (OT)

A 4.8 kb fragment extending from SpeI, 0.3 kb downstream of the polyadenylation signal, to KpnI, within the first intron of the adjacent vasopressin (VP) gene, was ligated into the XbaI-KpnI digested plasmid vector, pPNT. Next, a 2.4 kb SalI-SpeI fragment, whose 3′ terminus ended 0.5 kb upstream of the start codon, was subcloned into pBluescript II SK+ (Stratagene, La Jolla, CA), then transferred as a SalI-SalI fragment into the XhoI site of the modified pPNT plasmid. The resulting plasmid, pPNTΔOT, in which the entire coding sequence of the OT-neurophysin I gene was replaced by the gene for neomycin-resistance, was linearized and transfected into TC1 and D3 embryonic stem (ES) cells. At 3.5 days post coitum (dpc), C57BL/6 blastocysts were injected with 13–16 ES cells from clones positive for the null allele. The injected blastocysts were transferred to the uteri of pseudopregnant C57BL/6 X CBA F1 females (Jackson Laboratory, Bar Harbor, ME) at 2.5 dpc. Mice arising from germline transmission of the mutated ES genome were backbred 10 generations onto C57BL/6 for the current studies, with wild type C57BL/6 mice serving as controls.

### 6 hr Shift of mice and observation of labor

Eight week old OT knockout (OT KO) and wild-type (WT) mice were maintained on a 12 h∶12 h light:dark cycle with lights on from 06:00 until 18:00. On the evening of mating, females were placed with singly housed wild type males for three hours (17:30–20:30) and then checked for copulation plugs. At 3.5 days post coitum (dpc) the mice were placed in cages with running wheels, and their activity pattern was recorded. On the morning of dpc 4.5, to avoid disruption of implantation but also allow enough time for the shift to occur before labor, plugged mice were divided into three groups (shifted forward 6 hours, unshifted and shifted backward 6 hours). On this day, shifted forward mice experienced a six hour day followed by a 12 hour night and shifted backward mice experienced a 18 hour day followed by a 12 hour night. After this day, the light cycle continued on a 12:12 light:dark pattern until dpc 17. To avoid effects of masking on the timing of parturition, the mice were placed in constant darkness after the light cycle on day 17.5 dpc until parturition. No significant differences in pup number were observed between genotypes (data not shown). Activity patterns were recorded (Clocklab Actimetrics, Evanston, IL) similar to previous experiments ^1^. Each hour from 12:00 on day 18.5 dpc until parturition, the mice were observed using infrared goggles and the half-hour prior to the observation of separation first pup from the mother was recorded as the time of labor. Protocols were approved by the Animal Studies Committee of Washington University.

### Measurement of serum oxytocin in shifted and unshifted mice over 24 hour period on dpc 17.5

Eight week old WT mice were mated and left unshifted or subjected to a 6-h backward shift. To avoid effects of masking, the mice were placed in constant darkness after the light cycle on day 16.5 dpc until serum was collected. Blood was collected from 2–3 mice from the shifted or unshifted group at each of 8 time points over the circadian day (00:00, 03:00, 06:00, 09:00, 12:00, 15:00, 18:00 and 21:00) of dpc 17.5. Plasma concentration of oxytocin was determined by ELISA (Assay Designs, Ann Arbor Michigan) according to the manufacturer's instructions. Specificity for OT was confirmed by measurements in OT deficient mice (data not shown).
